# Obese dogs with and without obesity-related metabolic dysfunction – a proteomic approach

**DOI:** 10.1186/s12917-016-0839-9

**Published:** 2016-09-20

**Authors:** Asta Tvarijonaviciute, Jose J. Ceron, Carlos de Torre, Blanka B. Ljubić, Shelley L. Holden, Yann Queau, Penelope J. Morris, Josep Pastor, Alexander J. German

**Affiliations:** 1 Departament de Medicina i Cirugia Animals, Universitat Autónoma de Barcelona, 08193 Barcelona, Spain; 2 Interdisciplinary Laboratory of Clinical Pathology, Iterlab-UMU, Campus of Excellence Mare Nostrum, University of Murcia, Murcia, Spain; 3 Unidad de Proteómica, Hospital Clínico Universitario Virgen de la Arrixaca (HCUVA), IMIB-Arrixaca, 30120 Murcia, Spain; 4 Internal Diseases Clinic, Faculty of Veterinary Medicine, University of Zagreb, 10000 Zagreb, Croatia; 5 Department of Obesity and Endocrinology, University of Liverpool, Leahurst Campus, Chester High Road, Neston, Wirral CH64 7TE UK; 6 Royal Canin Research Center, B.P.4-650 Avenue de la Petite Camargue, 30470 Aimargues, France; 7 The WALTHAM Centre for Pet Nutrition, Freeby Lane, Waltham-on-the-Wolds, Melton Mowbray, LE14 4RT UK

**Keywords:** Antioxidants, Complement system, Dog, Immune response, Lipid metabolism, Metabolic syndrome, Obesity

## Abstract

**Background:**

Approximately 20 % of obese dogs have metabolic disturbances similar to those observed in human metabolic syndrome, a condition known as obesity-related metabolic dysfunction. This condition is associated with insulin resistance and decreased circulating adiponectin concentrations, but clinical consequences have not been reported. In order to define better the metabolic changes associated with obesity-related metabolic dysfunction (ORMD), we compared the plasma proteomes of obese dogs with and without ORMD.

A proteomic analysis was conducted on plasma samples from 8 obese male dogs, 4 with ORMD and 4 without ORMD. The samples were first treated for the depletion of high-abundance proteins and subsequently analysed by using 2-DE DIGE methodology.

**Results:**

Using mass spectrometry, 12 proteins were identified: albumin, apoliprotein A-I, C2, C3, C5, C4BPA, A2M, Uncharacterised protein (Fragment) OS = Canis familiaris, fibrinogen, IGJ, ITIH2, and glutathione peroxidase. In obese dogs with ORMD, the relative amounts of ten proteins (albumin, apoliprotein A-I, C2, C3, C5, C4BPA, A2M, Uncharacterised protein (Fragment) OS = Canis familiaris, fibrinogen, and ITIH2) were increased and two proteins (IGJ and glutathione peroxidase) were decreased, compared with obese dogs without ORMD. Specific assays were then used to confirm differences in serum albumin, apoliprotein A-I and glutathione peroxidase in a separate group of 20 overweight dogs, 8 with ORMD and 12 without ORMD.

**Conclusions:**

The current study provides evidence that, in obese dogs with ORMD, there are changes in expression of proteins involved in lipid metabolism, immune response, and antioxidant status. The clinical significance of these changes remains to be defined.

## Background

In humans metabolic syndrome (MetS) is an extensively studied entity that comprises obesity, hypertension, dyslipidaemia, and glucose intolerance [1], and is associated with the development of cardiovascular disease and type 2 diabetes [2]. The prevalence of metabolic syndrome in humans varies with age and ethnic groups, and it has been estimated that one-quarter of the world’s adult population has the MetS [1]. Recently, when human MetS criteria were adapted for dogs to define the condition of obesity-related metabolic dysfunction (ORMD), approximately 20 % of dogs with naturally occurring obesity were described as presenting with concurrent ORMD [3]. The presence of ORMD was associated with increased circulating insulin and decreased adiponectin concentrations, suggesting that ORMD might be associated with a range of metabolic disturbances. However, to date, no study has identified any direct association between these disturbances and clinical diseases of obese dogs, such that the pathophysiological significance of the changes that occur in ORMD remains unknown.

Proteomic analysis has been increasingly used in human medicine and experimental animals in order to evaluate possible changes in blood, adipose tissue, or adipocyte proteomes in obese subjects [4–7], with the aim of identifying and clarifying the underlying mechanisms responsible for development of secondary diseases. To the authors’ knowledge, the only proteomic study to date in canine obesity was performed under experimental conditions, and identified three proteins (retinol binding protein 4, clusterin, and α-1 antitrypsin) that differed between obese and lean dogs [3]. Whilst this study demonstrated proof of principle, it is possible to refine the technique reported and improve detection sensitivity by first depleting high-abundance protein species in the biological samples examined [8]. Such a depletion technique has been performed in some human proteomic studies of obesity [9, 10]. In addition, when comparing protein differences between two samples, fluorescence two-dimensional differential gel electrophoresis (2-DE DIGE) has shown benefits over conventional 2-DE methodology, since it enables two different samples to be run on the same gel in both dimensions thus minimising gel-to-gel variation [11, 12].

The main objective of the current study was to compare the plasma proteomes of obese dogs with and without ORMD in order to evaluate if the presence of ORMD imply changes in proteome of obese dogs.

## Methods

### Animals

The study protocol adhered to the University of Liverpool Animal Ethics Guidelines, and was approved by both the University of Liverpool Research Ethics Committee and the WALTHAM Animal Welfare & Ethical Review Board. All clinical procedures were performed for the direct benefit of the animals, and their owners gave informed written consent.

Plasma samples from eight neutered male obese dogs (group 1; 4 with ORMD, and 4 without ORMD) were used in the proteomic study. All dogs were originally referred to the Royal Canin Weight Management Clinic, University of Liverpool UK for investigation and management of obesity. Any surplus plasma, after that required for use in routine clinical biochemical analyses, was frozen at −20 °C, usually within 30 min of sample collection. All samples remained frozen until shipment on dry ice to the Interlab-UMU, Murcia, Spain, where all proteomic analyses were conducted.

In order to corroborate the proteomic analysis findings serum samples from a further 20 dogs (group 2) from different private veterinary clinics of Murcia Region, Spain, were used. Twelve dogs were overweight/obese without ORMD and eight overweight/obese with ORMD. These dogs were otherwise healthy as recorded on physical examination, CBC and biochemistry analyses. All procedures were approved by the Ethics Committees of University of Murcia and Murcia Region; in addition, an informed consent was obtained from the owner before inclusion of the dog in the study.

### Clinical measurements

Body condition score was determined using a validated 9-point body condition score system in both groups [13, 14]. In addition for group 1 dogs, body composition was analysed using fan-beam DEXA (Lunar Prodigy Advance; GE Lunar) [15–17]. Blood pressure in all dogs was measured non-invasively using an oscillometric method. All dogs were fully conscious. A cuff of the appropriate size (e.g. the cuff chosen had a width of ~40 % circumference of the leg) was placed on the right forelimb. Once the dog was calm and still, at least five systolic blood pressure (SBP) readings were taken and averaged. Finally, blood samples were collected by jugular venepuncture after a fast of at least 16 h.

### Definition of obesity-related metabolic dysfunction

Dogs were considered as having ORMD if the following criteria were met: (a) BCS 7-9/9; AND (b) any two of the following: 1) plasma triglycerides > 2.3 mmol/L; 2) plasma cholesterol > 7.8 mmol/L; 3) SBP > 160 mmHg; 4) fasting plasma glucose > 5.6 mmol/L, or previously diagnosed diabetes mellitus [3].

### Dog plasma pre-treatment (ProteoMiner)

Plasma samples from group 1 dogs were treated with ProteoMiner treatment according to manufacturer’s recommendation (BioRad Laboratories, Hercules, CA.). Briefly, 20 μL aliquots of ProteoMiner beads (small capacity kit) were equilibrated with washed buffer (phosphate buffer saline (PBS): 150 mM NaCl, 10 mM NaH_2_PO_4_, pH 7.4). Subsequently, 200 μL plasma samples were applied and incubated for 2 h at room temperature under constant rotation. After incubation, the beads were washed three times with 200 μL wash buffer to remove unbound proteins. Bound proteins were eluted with three washes of 20 μL elution reagent (8 M urea, 2 % CHAPS). Eluted proteins were cleaned using 2D clean-up kit (GE Healthcare Europe GmbH, Freiburg, Germany) and resuspended in rehydration buffer (30 mmol/L Tris basic, 2 mol/L thiourea, 7 mol/L urea, 4 % wt/vol CHAPS).

### Protein sample preparation and fluorescence labelling

Protein concentration was determined with 2Dquant kit (GE Healthcare Europe GmbH, Freiburg, Germany). The DIGE fluors –N-hydroxysuccinimidyl ester derivatives of the cyanine dyes, Cy2, Cy3 and Cy5 (GE Healthcare Europe GmbH, Freiburg, Germany) –were prepared using freshly opened N,N-dimethylformamide (Sigma-Aldrich). Protein samples (50 μg) were labelled with 400 pmol of Cy2, Cy3 and Cy5 DIGE fluors in separate tubes on ice for 30 min. An internal standard was created by mixing equal amounts of proteins of all samples and labelling it with Cy2, whereas individual samples were labelled with Cy3 and Cy5. The labelling reaction was quenched by adding 1 μL of 10 mmol/L lysine.

### Two-Dimensional DIGE

The immobilised pH gradient strips (pH 4–7; GE Healthcare Europe GmbH, Freiburg, Germany) were actively rehydrated for 16 h according to the instructions of the manufacturer. The Cy2-labelled internal standard of pooled samples was used to minimise experimental gel-to-gel variation and to facilitate cross-gel quantitative analysis. Equal amounts (50 μg protein per dye per gel) of Cy3- and Cy5-labelled individual samples and the Cy2-labeled internal standard were mixed together and loaded onto an 18-cm immobilised pH gradient strip (pH 4–7). The proteins were focused in the first dimension for a total of 60000 Vh in the Ettan IPGphor 3 system (GE Healthcare Europe GmbH, Freiburg, Germany). The immobilised pH gradient strips were equilibrated first in buffer I [6 mol/L urea, 2 % SDS, 75 mmol/L Tris (pH 8.8), 30 % glycerol, 1 % DTT] for 15 min and then in buffer II (same as buffer I plus 2.5 % iodoactamide) for 15 min and subsequently transferred onto 12.5 % gels. Proteins were then separated in the second dimension based on their molecular weight in the Ettan DALT six (GE Healthcare Europe GmbH, Freiburg, Germany). A total of six SDS gels were run together at 5 mA per gel for 2 h and 2 W per gel for 12 h with the temperature of the running buffer maintained at 23 °C.

### Image acquisition and analysis

Gels were scanned directly between glass plates at 488/520 nm, 532/580 nm and 633/670 nm (excitation/emission wavelengths) for the Cy2, Cy3 and Cy5 signals, respectively, using a Typhoon 9410 fluorescent scanner (GE Healthcare Europe GmbH, Freiburg, Germany). Raw data of images were analysed with DeCyder 7.0 software (GE Healthcare Europe GmbH, Freiburg, Germany). Briefly, original images of Cy2, Cy3 and Cy5 images for each gel were cropped and smoothed to clarify spots. The software was used following manufacturer recommendations, and the estimated number of spots was set to 1500. The internal standard image with the most detected spots was assigned as the master and it was selected to create a MatchSet image. A MatchSet image was created for comparing and analysing spots from all 11 images and a master image. The Cy2 spot data from each gel were normalised on a spot-to-spot basis using respective Cy3 and Cy5 signals of the internal standard from the same gel, and the normalised Cy2 signal was then used for spot intensity comparison and analysis. The protein abundance for each spot in each sample was then expressed as a ratio relative to the internal standard. Statistical analysis was performed for every matched spot set, comparing the average ratio for a given spot between groups. Only those spots with a *p* < 0.05 and a fold change greater than 1.5 and lower than −1.5 were considered.

### Preparatives 2D gel

Preparative gel, containing 275 μg of protein, were prepared for a mix used as internal standard using PlusOne™ Bind-Silane following manufacturer recommendations (GE Healthcare Europe GmbH, Freiburg, Germany). These gels were fixed and stained with Colloidal Coomassie G-250 following the previously described protocol [18]. The images were then captured using the Image Scanner III System (GE Healthcare Europe GmbH, Freiburg, Germany). The desired protein spots were picked from the Coomassie stained gels with Ettan Spot Picker (GE Healthcare Europe GmbH, Freiburg, Germany).

### Protein identification

Proteins within gel spots were first reduced and alkylated using DTT and iodoacetamide, respectively, and then digested to peptides by trypsin proteomics grade (Sigma-Aldrich, St Louis, MO, USA) [19].The tryptic peptides were analysed by capillary reverse-phase liquid chromatography coupled online with MS/MS. The column, BioBasic-18, 5 μm particles, 300 Å pore size, 0.18 mm ID-100 mm L (Thermo, San Jose, CA), was connected to a Surveyor MS Pump Plus (Thermo, San Jose, CA) and then coupled with an ion trap mass spectrometer (LXQ, Thermo, San Jose, CA). The flow rate was set at 180 μl/min but split to a flow rate of 2 μl/min. Mobile phase A was 0.1 % formic acid/2 % methanol in water and B was 0.1 % formic acid in methanol. The peptide samples were injected and gradient elution was done under the following conditions: 5 % B in 2 min; a linear increase of 5 to 70 % B in 20 min; 70 % for 10 min; 5 % B for 10 min. The ion trap MS was operated in a data-dependent MS/MS mode where the five most abundant peptide molecular ions in every MS scan were sequentially selected for collision-induced dissociation with a normalised collision energy of 34 %. Dynamic exclusion was applied to minimise repeated selection of peptides previously selected for collision induced dissociation. The capillary temperature and electrospray voltage were set to 200 °C and 3.5 kV, respectively. The resulting mass spectra were searched against the *Canis familiaris* Uniprot database (26456 sequences, released at September 19, 2013) with the Proteome Discoverer 1.3 software (Thermo, San Jose, CA). The following search parameters were applied: default charge states of 2+, 3+, and 4+ were used; a maximum of one missed cleavage was allowed with an average peptide mass tolerance of 1.5 Da. A fragment ion search tolerance of 0.8 Da was permitted. Fixed modification on cysteine was carbamidomethylation and a variable modification was oxidation of methionine. All data were searched against a decoy database. The peptide concentration score cut-off for each of the runs was automatically adjusted to ensure a 1 % false discovery rate throughout the experiments. A positive identification was assigned when two or more unique peptides were identified.

### Verification of proteomic analysis by specific assays

Serum samples from group 2 dogs were analysed for albumin (Albumin, Beckman Coulter Ireland Inc., Lismeehan, Ireland), apoliprotein A-1 (Apoliprotein A-I (APO A-I), BioSystems, Barcelona, Spain), and glutathione peroxidase (Ransel, Randox Laboratories Limited, Crumlin, United Kingdom). All the methods were performed in automated analyzer (Olympus AU600, Beckman Coulter, Brea, USA). All assays showed an inter- and intra-assay imprecision less than 15 % and a linearity under dilution resulted in coefficient of correlation close to 1 with canine serum samples.

### Statistical analysis

Statistical analysis was performed using routine descriptive statistical procedures and software (Graph Pad Prism, Version 5). In order to compare different variables between dogs with and without ORMD of the group 2, the data were first evaluated for normality of distribution using a D’Agostino & Pearson omnibus normality test, giving not parametric distribution. For this, Mann–Whitney test was performed. Values of *P* < 0.05 were considered significant.

## Results

### Comparison of obese dogs with and without ORMD

The baseline characteristics of group 1 dogs with and without ORMD (participating in proteomic analyses) are shown in Table 1, with no marked differences between the groups, in terms of age, breed, weight, and body fat percentage. All 4 dogs with ORMD had increased SBP, whilst 3, 3, and 2 dogs, respectively, had increased glucose, cholesterol, and triglyceride concentrations.

**Table 1:** The baseline characteristics of dogs included in proteomic study with and without obesity-related metabolic dysfunction (ORMD)

Variable	ORMD	No ORMD
Sex	4 males (all neutered)	4 males (all neutered)
Age, months	60 (25–120)	70 (43–120)
Breed	Labrador Retriever (2), Golden Retriever; Patterdale terrier	Border Terrier
		Labrador
		Mixed Breed
		Newfoundland
Weight, kg	36.7 (13.8–100.0)	49.6 (25.8–60.8)
Total BodyFat, %	52.4 (50.8–60.8)	52.1 (44.4–55.1)
BCS	9 (9–9)	9 (7–9)
SBP, mmHg	174 (166–177)	141 (123–159)
Glucose, mmol/L	5.6 (5.1–6.0)	5.3 (4,8–5,5)
Cholesterol, mmol/L	6.9 (4.6–8.0)	5.9 (4,9–6,1)
TG, mmol/L	1.3 (0.8–2.3)	0.8 (0.5–1.2)

Data are presented as median (interquartile range)

*BCS* body condition score, *SBP* systolic blood pressure, *TG* triglycerides

The baseline characteristics of group 2 dogs the animals with and without ORMD (used for assay verification) are shown in Table 2. Again, there were no marked differences between the groups, in terms of age, breed, and weight. Of the 8 dogs with ORMD, five had hypercholesterolaemia, four had hypertriglyceridaemia, and six had hyperglycaemia. Of the 12 dogs without ORMD, two had hypercholesterolaemia, one had hypertriglyceridaemia, and two had hyperglycaemia.

**Table 2:** The baseline characteristics of dogs included in proteomic study verification with and without obesity-related metabolic dysfunction (ORMD)

Variable	ORMD	No ORMD	*P*
Number	8	12	
Sex	3 males and 5 females (all neutered)	5 males and 7 females (all neutered)	
Age, months	100 (36–139)	81 (24–132)	NS
Breed	Mixed Breed (2),	Mixed Breed (7)	
	Boxer,	Yorkshire terrier (2)	
	Beagle,	Bichon Maltese	
	Brittany Spaniel	Collie	
	Cocker Spaniel,	Shih tzu	
	French Bulldog,		
	Yorkshire terrier,		
Weight, kg	12.4 (11.4–18.7)	20.3 (5.2–44.5)	NS
BCS	9 (8–9)	9 (7–9)	NS
SBP, mmHg	140 (123–150)	130 (110–158)	NS
Glucose, mmol/L	5.8 (5.7–7.6)	4.9 (4.7–5.3)	0.047
Cholesterol, mmol/L	9.1 (7.6–11.0)	5.8 (5.2–7.6)	0.007
TG, mmol/L	2.8 (0.8–3.8)	0.9 (0.6–1.4)	NS

Data are presented as median (interquartile range)

*BCS* body condition score, *SBP* systolic blood pressure, *TG* triglycerides, *NS* not statistically significant

### Proteomic analysis

**Fig. 1:**
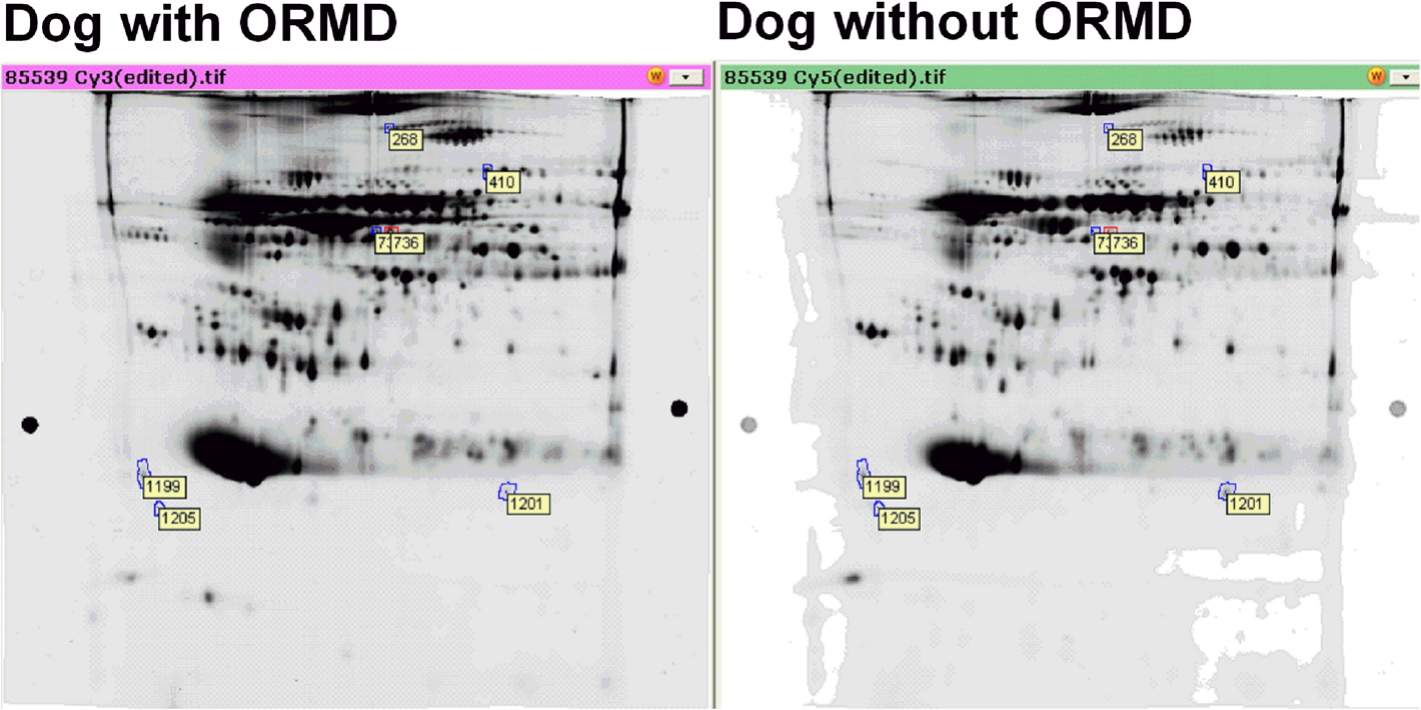
Representative 2-DE gel images proteins of ProteoMiner pre-treated plasma samples obtained from dogs with and without obesity-related metabolic dysfunction (ORMD). Differentially expressed protein spots between the dogs with and without ORMD are circled and numbered

**Fig. 2:**
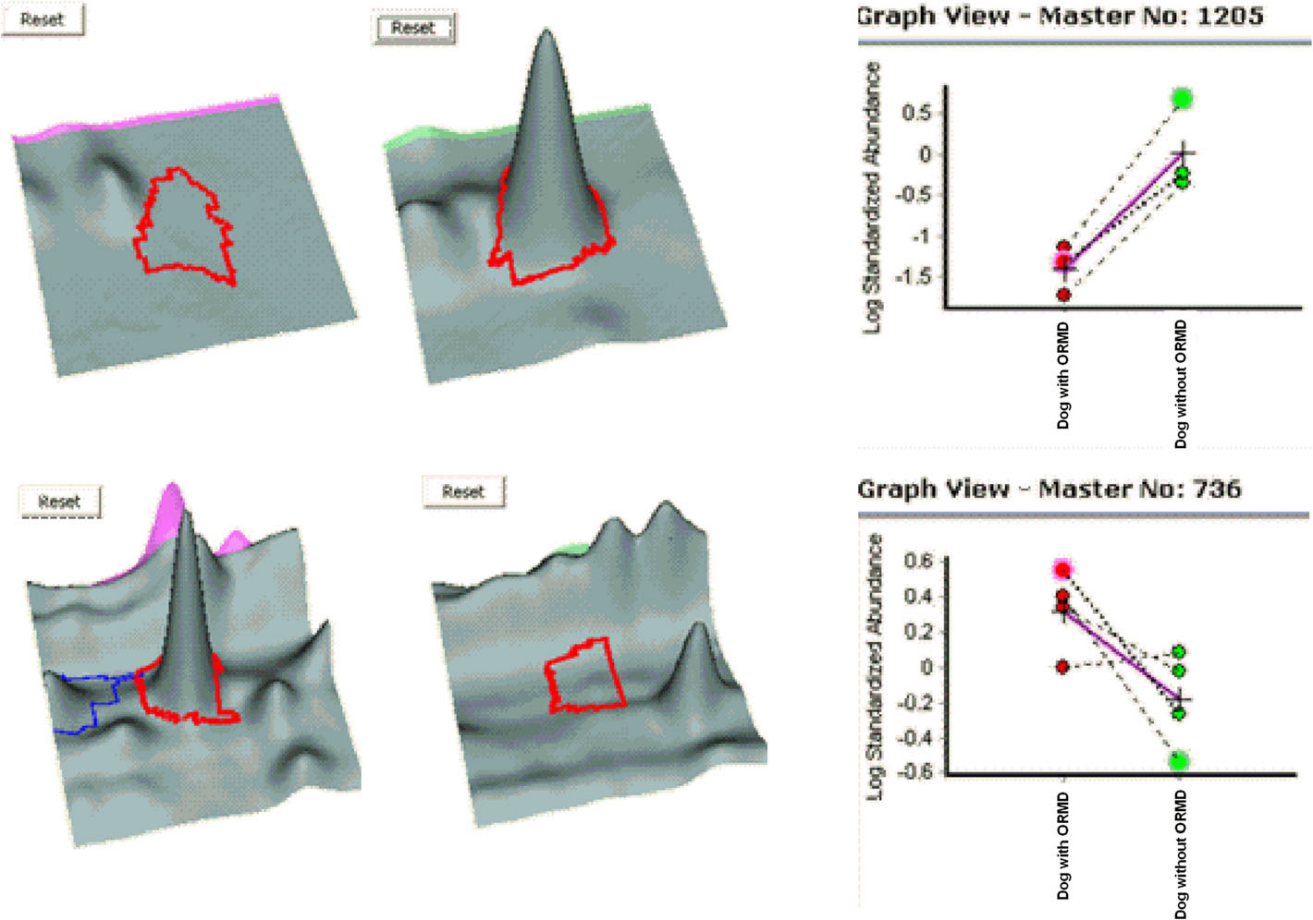
Example of the 3-dimensional images of differentially regulated spot proteins (one increased and one decreased) in dogs with (left) and without (right) obesity-related metabolic dysfunction (ORMD). Graphics represent the changes in Standardised Abundance of specific spots after logarithmic transformation obtained directly from DeCyder 7.0 software analysis: dotted lines represent individual data, entire lines represent mean

More than 1200 individual spots, ranging from 15 to 250 kDa masses over pH 4–7 were detected in gels of protein mapping of canine plasma pre-treated with ProteoMiner. Using image analysis and further statistical analysis, 8 spots were detected and subsequently identified with differential protein concentrations between dogs with and without ORMD (Fig. 1). Among these 8 protein spots, protein concentrations were increased in 3 and decreased in 5 obese dogs with ORMD compared with obese dogs without ORMD (Table 3, Fig. 2).

**Table 3:** Fold change of differentially expressed spot matches in dogs with (*n* = 4) and without (*n* = 4) obesity-related metabolic dysfunction (ORMD)

Spot^a^	Fold change noORMD dogs/ORMD dogs	*P*
1205	42.68	0.017
1201	2.55	0.005
1199	1.53	0.006
410	−1.76	0.040
268	−2.08	0.041
730	−2.28	0.036
624	−2.39	0.009
736	−3.09	0.031

^a^Spot label number from annotated gel image (see Figs. 1 and 2)

### Mass spectrometry

Mass spectrometry allowed the identification of 12 proteins from seven out of eight spots detected (Table 4), which were identified as: alpha-2 macroglobulin (A2M) and, apoliprotein A-I (spot number 268), C2 and C5(spot 410), fibrinogen and Uncharacterised protein (Fragment) OS = Canis familiaris (spot 624), albumin (spot 730), C3 (spots 730, 736 and 1190), ITIH2 (spots 730. 736 and 1190), C4BPA (spot 1190), IGJ(spot 1199), and glutathione peroxidase (spot 1201). Unfortunately, the proteins in spot 1205 could not be identified. Among these 12 proteins, concentrations were increased in 10 and decreased in 2 in obese dogs with ORMD compared with obese dogs without ORMD. The proteins with increased expression in ORMD were: albumin, apoliprotein A-I, C2, C3, C5, C4BPA, A2M, Uncharacterised protein (Fragment) OS = Canis familiaris, fibrinogen, and ITIH2, whilst the two proteins with decreased expression in ORMD were: IGJ and glutathione peroxidase.

**Table 4:** Mass spectrometry identification of proteins

Spot No^a^	Protein name	Accession number^b^	Score	Unique peptides	Sequence coverage (%)^c^	MW (kDa)/pI
268	Uncharacterised protein *Canis familiaris* (A2M)	F6UME0	99.79	10	8.14	165.1/6.71
	Apolipoprotein A-I *Canis familiaris*	F1PDJ5	11.42	2	9.40	30.2/5.39
410	Uncharacterised protein *Canis familiaris* (C2)	E2RS75	132.97	9	16.56	86.3/7.37
	Uncharacterised protein *Canis familiaris* (C5)	F1P7J4	18.61	2	1.36	188.5/6.60
624	Fibrinogen alpha chain OS = *Canis familiaris*	J9NRV7	112.52	5	7.05	88.9/6.16
	Uncharacterised protein (Fragment) OS = *Canis familiaris*	F1PWR2	12.25	3	2.45	192.8/6.89
730	Uncharacterised protein *Canis familiaris* (C4BPA)	F1PGM9	390.27	11	30.93	57.9/6.83
	Albumin *Canis familiaris*	F2Z4Q6	174.32	8	15.30	68.6/5.69
	Uncharacterised protein *Canis familiaris* (C3)	F1PGM1	64.87	5	2.81	176.0/7.12
	Uncharacterised protein *Canis familiaris* (ITIH2)	F1PG39	4.96	3	4.96	107.2/7.53
736	Uncharacterised protein *Canis familiaris* (C4BPA)	F1PGM9	456.80	8	23.93	57.9/6.83
	Uncharacterised protein *Canis familiaris* (ITIH2)	F1PG39	237.71	6	11.39	107.2/7.53
	Uncharacterised protein *Canis familiaris* (C3)	F1PGM1	128.64	11	8.62	176.0/7.12
	Albumin *Canis familiaris*	F2Z4Q6	38.31	2	5.59	68.6/5.69
1199	Uncharacterised protein *Canis familiaris* (IGJ)	J9JHH5	43.95	2	12.58	18.2/4.82
1201	Glutathione peroxidase (Fragment) *Canis familiaris*	J9P028	322.78	4	37.91	17.3/7.97

*MW* molecular weight

^a^Spot label number from annotated gel image (see Figs. 1 and 2). Spot No 1205 Not identified

^b^UniProtKB database. *Canis familiaris*(26456 sequences, 15304077 residues, 09/19/2013)

^c^Sequence coverage: percentage of identified sequence to the complete sequence of the known protein

### Verification of proteomic analysis by specific assays

**Fig. 3:**
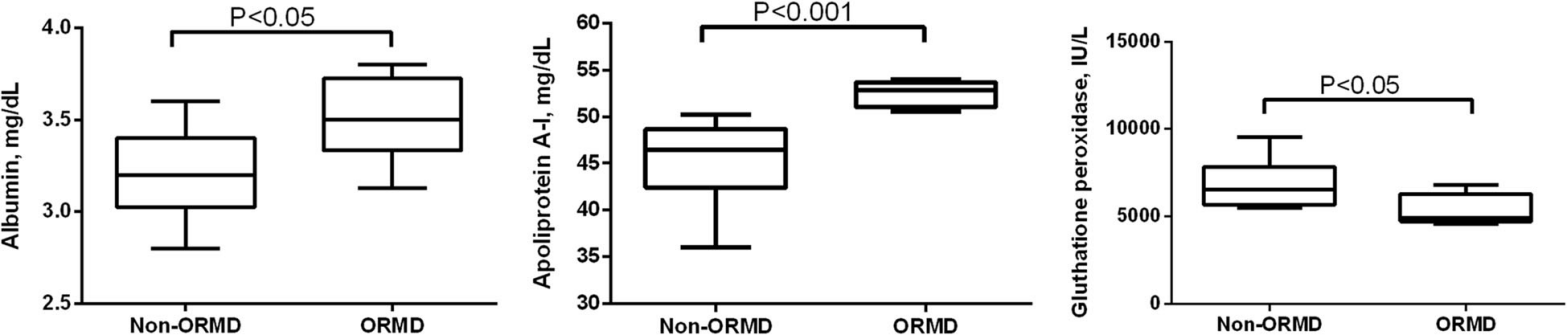
Serum albumin, apoliprotein A-I, and gluthatione peroxidase in dogs with (ORMD; *n* = 8) and without (Non-ORMD; *n* = 12) obesity related metabolic dysfunction (ORMD)

Specific assays were used to verify the results of proteomic analyses in a separate group of dogs. Plasma albumin (*P* = 0.044) and apoliprotein A-I (*P* < 0.001) concentrations were greater in dogs with ORMD, and there was also decreased activity of glutathione peroxidase (*P* = 0.039) (Fig. 3).

## Discussion

The aim of the present study was to evaluate differences in the plasma proteome of obese dogs with and without ORMD. For this, we used 2D DIGE and mass spectrometry to identify a set of proteins that differed significantly in abundance in the plasma of obese dogs with ORMD relative to obese dogs without ORMD. In total, 12 differentially expressed proteins were identified. These were proteins associated with lipid metabolism (albumin, apoliprotein A-I), the complement system (C2, C3, C5, C4BPA, A2M, Uncharacterised protein (Fragment) OS = Canis familiaris), coagulation (fibrinogen), immune response (IGJ), hyaluronan metabolic process (ITIH2), and antioxidants (glutathione peroxidase). These findings provide further evidence that ORMD in dogs is associated with a range of metabolic changes, although their clinical significance remains unknown.

Increased albumin abundance was observed in the plasma of obese dogs with ORMD compared with dogs without ORMD by both proteomic and specific analysis. Significantly greater albumin concentrations have previously been reported in obese compared with healthy dogs [20], and circulating concentrations of albumin are known to decrease after weight loss [17, 20]. However, not all studies have identified such differences [21, 22]. That said, increased albumin expression has been detected in the proteome of mature adipocytes collected from overweight and morbidly obese persons [7]. Further studies would be indicated in order to determine the mechanisms involved for the increased circulating albumin in obese dogs with ORMD.

Apolipoprotein A1 (APO-A1) participates in the reverse transport of cholesterol from tissues to the liver, ready for its subsequent excretion. APO-A1 has also been reported to have acyl coenzyme A cholesterol acyltransferase-like activity; this may synergistically regulate adipogenesis and lipid metabolism [23]. In the current study, greater APO-A1 concentrations were detected in dogs with ORMD as observed in proteomic analysis and verified by a specific assay. These results are consistent with those reported by Briand et al. [24] who, in a stable isotope study, observed increased APO-A1 production in insulin resistant dogs.

In the present study, six different proteins (C2, C3, C5, C4BPA, A2M, Uncharacterised protein (Fragment) OS = *Canis familiaris*) associated with the complement arm of the immune system were higher in abundance in dogs with ORMD. In two human proteomics studies, plasma C3 was identified as a key marker for differences in body fat and body fat changes [10, 27]. Further, in humans C3 was associated with the prevalence of MetS and diabetes mellitus type 2 development [25]. It was speculated that increased concentrations of proteins of complement system could be due to decreased activity of the complement system or due to changes in production in adipose tissue or at other sites [10, 26]. Similar mechanisms might be responsible for the findings in obese dogs with ORMD, but more work would be required to clarify this.

Dogs with ORMD also had greater fibrinogen concentrations than dogs without ORMD. It has been suggested fibrinogen to be an additional defining component of metabolic syndrome in humans [28], as fibrinogen concentrations were increased in subjects with metabolic syndrome compared with those without it [28, 29]. It has been hypothesised that fibrinogen acts as a link between prothrombotic and proinflammatory states in metabolic syndrome in humans [28].

J-chain of immunoglobulin (IGJ) is a protein of about 15 kDa polypeptide, expressed by mucosal and glandular plasma cells. It regulates polymer formation of immunoglobulin (Ig) A and IgM. In humans increased IgA concentration is associated with components of metabolic syndrome, such as hyperglycaemia, hypertriglyceridaemia, and abdominal obesity [30]. It has been suggested that increased IgA and IgM concentrations could be attributed to the presence of low-grade inflammation in obesity [30]. Taken together, it could be hypothesised that lower abundance of IGJ observed in dogs with ORMD in present study, could be attributed to its greater utilisation by IgA or/and IgM. However, future studies are required in order to clarify this.

The inter-alpha (globulin) inhibitor (ITI) family (more commonly called the family of inter-alpha-trypsin inhibitors) is composed of serine protease inhibitors that are assembled from two precursor proteins: a light chain and either one or two heavy chains [31, 32]. Whilst there is only one type of light chain, to date, five different homologous heavy chains (ITIHs) have been identified in this family (ITIH1-ITIH5) [33]. The only currently known function of the heavy chains is to bolster the stability of extracellular matrix through covalent linkage to hyaluronic acid [34], which is a major component of this matrix [33].A number of studies have reported on the biological effects of the ITI molecules, proposing involvement in various acute-phase processes, such as inflammation or cancer [33, 35]. ITIH2 was shown to be down-regulated in tumours of the breast, compared with other ITIH proteins, suggesting its potential function as tumour suppressor or metastasis repressor [33]. In the present study, increased abundance of ITIH2 was observed in obese dogs with ORMD and, to the authors’ knowledge, no previous studies have linked this protein to metabolic syndrome.

Glutathione peroxidase is an 80 kDa protein, which is composed of four identical subunits, and acts as a major antioxidant in the plasma [35]. Decreased glutathione peroxidase abundance was observed by proteomic study in obese dogs that presented with ORMD. These findings were also confirmed by use of a specific assay. During the development of obesity, glutathione peroxidase precursor expression decreases in obesity-prone rats, but increases in both obesity-resistant and normal rats [35]. It was hypothesised that these changes could lead to augmented systemic oxidative stress and the onset of metabolic complications such as diabetes mellitus [36].

The main limitation of the present study was the relatively small number of animals studied by proteomic analysis. In light of this, only samples from male dogs were used to perform proteomic analysis, so there could be no possible confounding effect of sex or/and hormones. Previous studies have suggested that three biological samples are sufficient to detect induced biological variations in the proteome of animals [37, 38]. A second potential limitation of the present study relates to storage of the samples used for proteomic analysis prior to their transportation to InterLab-UMU, Spain. It is possible that such storage may have affected the results, for instance by resulting in fewer spots/proteins being detected. Thirdly, the included animals were client-owned dogs with variable living conditions and different diets, what could have influenced the results of the study. Nevertheless, the data are representative of a true clinic picture. Despite these limitations and, although ideally all identified proteins should have been validated, the verification of three proteins agrees with the data observed by proteomic study.

## Conclusions

The results of this comparative plasma proteome analysis have demonstrated that the obese dogs with ORMD have alterations in the concentrations of proteins related to lipid metabolism, immune response, and antioxidant system. Further studies should be performed to evaluate the clinical implications that changes in these proteins could have.

## Abbreviations

2-DE DIGE: Two-dimensional differential gel electrophoresis
A2M: Alpha-2-macroglobulin
APO-A1: Apoliprotein A-I
BCS: Body condition score
C2: Complement component 2
C3: Complement component 3
C4BPA: Complement component 4 binding protein alpha
C5: Complement component 5
DEXA: X-ray absorptiometry dual energy
IgA: Immunoglobulin A
IGJ: J-chain of immunoglobulin
IgM: Immunoglobulin B
ITIH: Inter-alpha-trypsin inhibitors
ORMD: Obesity related metabolic dysfunction
PBS: Phosphate buffer saline
SBS: Systolic blood pressure.
